# In Absence of the Cellular Prion Protein, Alterations in Copper Metabolism and Copper-Dependent Oxidase Activity Affect Iron Distribution

**DOI:** 10.3389/fnins.2016.00437

**Published:** 2016-09-27

**Authors:** Lisa Gasperini, Elisa Meneghetti, Giuseppe Legname, Federico Benetti

**Affiliations:** Laboratory of Prion Biology, Department of Neuroscience, Scuola Internazionale Superiore di Studi AvanzatiTrieste, Italy

**Keywords:** prion, copper, iron, oxidase activity, essential metals

## Abstract

Essential elements as copper and iron modulate a wide range of physiological functions. Their metabolism is strictly regulated by cellular pathways, since dysregulation of metal homeostasis is responsible for many detrimental effects. Neurodegenerative disorders such as Alzheimer's disease, Parkinson's disease and prion diseases are characterized by alterations of metal ions. These neurodegenerative maladies involve proteins that bind metals and mediate their metabolism through not well-defined mechanisms. Prion protein, for instance, interacts with divalent cations via multiple metal-binding sites and it modulates several metal-dependent physiological functions, such as S-nitrosylation of NMDA receptors. In this work we focused on the effect of prion protein absence on copper and iron metabolism during development and adulthood. In particular, we investigated copper and iron functional values in serum and several organs such as liver, spleen, total brain and isolated hippocampus. Our results show that iron content is diminished in prion protein-null mouse serum, while it accumulates in liver and spleen. Our data suggest that these alterations can be due to impairments in copper-dependent cerulopalsmin activity which is known to affect iron mobilization. In prion protein-null mouse total brain and hippocampus, metal ion content shows a fluctuating trend, suggesting the presence of homeostatic compensatory mechanisms. However, copper and iron functional values are likely altered also in these two organs, as indicated by the modulation of metal-binding protein expression levels. Altogether, these results reveal that the absence of the cellular prion protein impairs copper metabolism and copper-dependent oxidase activity, with ensuing alteration of iron mobilization from cellular storage compartments.

## Introduction

Biometals are essential for a wide range of functions in living organisms. They are involved in a variety of cellular pathways, from electrochemical gradient generation to enzyme active site formation, DNA replication, oxygen transport, and oxidative/nitrosative stress regulation. Metal absorption, metabolism, and excretion are tightly regulated processes. Although ion-specific transporters and metal-binding proteins exist, absorption/excretion pathways are highly interconnected and interdependent among different ionic species. In particular, these interactions occur at two levels: (i) sharing of some common transporters, e.g., divalent metal transporter 1; (ii) existence of enzymes involved in the homeostasis of several essential metals, for instance the copper-dependent ferroxidase ceruloplasmin (Cp). Hence, the impairment of metal homeostasis could affect the metabolism of a variety of processes (Arredondo and Nunez, 2005; Kambe et al., 2008; Bleackley and Macgillivray, 2011). Because of their chemistry, transition metals, present in living organisms, need to be properly compartmentalized and distributed to avoid detrimental effects, such as reactive oxygen species (ROS)/reactive nitrogen species (RNS) generation and abnormal metal-protein interaction. Indeed, metal homeostasis dysregulation is highly damaging and could result in developmental abnormalities and defects, neurodegeneration or early death (Bush, 2000; Hellman and Gitlin, 2002; Zecca et al., 2004; Gaier et al., 2013). Biometal homeostasis alterations are closely related to neurodegenerative mechanisms, mainly through two reactions: (i) aberrant metal–protein association leading to protein aggregation and/or altered enzyme activity; (ii) metal-catalyzed protein oxidation leading to protein damage and denaturation (Bush, 2000). Several disorders show a dysmetabolism of metal ions, such as Menkes's and Wilson's disorders, aceruloplasminemia, Friedreich's ataxia, amyotrophic lateral sclerosis, schizophrenia, Alzheimer's disease, Parkinson's disease, and prion diseases (Bush, 2000; Hellman and Gitlin, 2002; Zecca et al., 2004; Gaier et al., 2013). Some of these illnesses are due to mutations in well-defined metal-binding proteins, while Alzheimer's disease, Parkinson's disease and prion diseases are protein aggregation disorders in which amyloid precursor protein, α-synuclein and prion protein, respectively, bind metals and are involved in metal metabolism with no well-defined physiological functions.

Prion diseases are a class of neurodegenerative disorders characterized by aggregation of an endogenous metal-binding protein, the cellular prion protein (PrP^C^), which undergoes a conformational change into a β-sheet enriched isoform, the scrapie prion protein (PrP^Sc^) (Prusiner, 2001). PrP^C^, encoded in mouse by the *Prnp* gene, is a glycosylphosphatidylinositol (GPI)-anchored protein widely expressed in the organism with the highest level in the central nervous system (CNS), in particular in the hippocampus (Horiuchi et al., 1995; Fournier et al., 1998; Ning et al., 2005; Benvegnu et al., 2010). In the unfolded N-terminal domain, PrP^C^ contains the octapeptide region (OR) which binds with high affinity copper, supporting its reduction from Cu(II) to Cu(I), and with lower affinity manganese and zinc (Brown et al., 1997; Jackson et al., 2001; Miura et al., 2005).

Results from PrP^C^-null (*Prnp*^0/0^) mice suggest a cellular role for PrP^C^ in which metals are crucial factors, such as their involvement in antioxidant defense, neuroprotection against excitotoxicity, myelin formation, and maintenance (Rangel et al., 2007; Khosravani et al., 2008; Bremer et al., 2010). In particular, we have recently shown that PrP^C^-copper complex modulates NMDA receptor by S-nitrosylation (Gasperini et al., 2015). Moreover, PrP^C^ seems to be critical for metal homeostasis (Brown, 2003; Singh et al., 2009c; Watt et al., 2012). Concerning copper, PrP^C^ has been proposed to pass it to transporters, and also to buffer and redistribute it at synapses after its release during synaptic transmission (Vassallo and Herms, 2003).

In this work, we focused our attention on the effect of PrP^C^ ablation on copper and iron metabolism. Copper and iron are key neurochemical factors whose aberrant interactions with target proteins could induce reactions relevant for pathophysiology of neurodegeneration. Copper is an essential metal for which PrP^C^ has the highest binding affinity (Lutsenko and Petris, 2003), and iron is the most abundant trace element (Schroeder and Nason, 1971) whose metabolic pathways are highly dependent on copper (Ganz, 2013). Since essential metals play key roles throughout development and exert pleiotropic, metal-specific, and often cell-specific effects on morphogenesis, growth, and differentiation, we carried out our study by comparing *Prnp*^+/+^ and *Prnp*^0/0^ mice at different ages, from early post-natal days to 1-year-old. In particular, we investigated copper and iron content, their distribution and functional value as well as the genetic and physiology of essential metal-binding proteins. Here, we show that the absence of PrP^C^ impairs copper metabolism and copper-dependent oxidase activity, thus affecting iron mobilization.

## Experimental procedures

### Materials

Except where specified, all chemicals were obtained from Sigma-Aldrich (St Louis, MO, USA). Protease inhibitor cocktail was from Roche Diagnostics Corp. (Mannheim, Germany). ECL detection reagent was from GE Healthcare (Waukesha, WI, USA). Anti-Ctr1 ab123105, anti-Sod1 ab13499 were from Abcam (Cambridge, UK). Recombinant anti-PrP^C^ humanized Fab D18 ABR-0D18 was purchased from InPro Biotechnology (South San Francisco, CA, USA). Monoclonal anti-PrP SHA31 A03213 was from BertinPharma (Montigny le Bretonneux, France). Anti-TfR1 13-6800 was from Invitrogen (Paisley, UK). Anti-β-Tubulin III T2200, monoclonal anti-β-Actin Peroxidase (AC-15) A3854, anti-Steap3 AV43515, anti-Atp7a AV33797, anti-human Ferritin F5012 were from Sigma-Aldrich. Anti-Ccs (FL-274) sc-20141, anti-FtH (H-53) sc-25617 were from Santa Cruz Biotechnology (CA, USA). Anti-Cp 611488 was from BD Transduction Laboratories (Milan, Italy). Anti-Tf GTX21223 was from GeneTex (Texas, USA). Anti-Fpn1 NBP1-21502 was from Novus Biologicals (Littleton, CO, USA). The anti-Atp7b N-WNPD#1 was kindly provided by Prof. S. Lutsenko, Johns Hopkins University, Baltimore, MD, USA. *O*-dianisidine dihydrochloride D3252 was from Sigma-Aldrich. The ELISA kit for Hepdicin SEB979Mu was from USCN (UK).

### Animals

All experiments were performed in accordance with European regulations [European Community Council Directive, November 24, 1986 (86/609/EEC)]. Experimental procedures were notified to and approved by the Italian Ministry of Health, Directorate General for Animal Health. All experiments were approved by the local authority veterinary service of Trieste, Italy, and by the Ethics Committee of the Scuola Internazionale Superiore di Studi Avanzati (SISSA), Trieste. All efforts were made to minimize animal suffering and to reduce the number of animals used. Inbred FVB/N (Friend virus B-type susceptibility-NIH) wild-type and FVB *Prnp*^0/0^ mice were used in these experiments. The FVB *Prnp*^0/0^ mice were obtained from George A. Carlson, McLaughlin Research Institute, Great Falls, Montana, and were bred by backcrossing with the original *Prnp*^0/0^ mice at least 20 times (Lledo et al., 1996).

### Metal ion measurements

Metal ion content was measured in: serum, liver and spleen collected from male mice at P15 (postnatal day 15), P30, P90, P180, and P365; total brain and isolated hippocampus collected from male mice at P1, P7, P30, P90, P180, P365. Liver and spleen were extracted after animal perfusion with physiological saline (0.9% NaCl) to minimize blood contamination, while brains and hippocampi were extracted and dissected in bidistilled water (ddH_2_O). To perform perfusion, animals were deeply anesthetized with urethane (2.5 g/kg). To minimize variations, all adult animals were perfused with the same saline volume, while a lower volume was used for P15 mice. Samples were completely dried in vacuum conditions and dissolved in 5 volumes of 65% nitric acid overnight at 70°C, except P1 and P7 hippocampi which were dissolved in 10 volumes. To prepare serum samples, the blood was collected by cardiac puncture from deeply anestethized animals and centrifuged for 10 min, RT at 900 × g. To measure ceruloplasmin-bound copper, serum samples were dialyzed against 50 mM Tris-HCl, 20 mM EDTA, pH 7.5, using Slide-A-Lyzer MINI Dialysis Device, 7K MWCO, 0.1 mL (69560, Thermo Scientific, MA, USA) (Favier and Ruffieux, 1988; Linder and Hazegh-Azam, 1996). An atomic absorption spectrophotometer (AAnalyst 100, PerkinElmer, MA, USA) was used to measure copper and iron in liver, spleen and serum. Inductively coupled plasma mass spectroscopy (ICP-MS, NexION 300D, PerkinElmer) was used to measure copper and iron in total brains and isolated hippocampi.

### Western blot

Protein levels were measured in liver, serum, total brain, and isolated hippocampus extracted from male mice at the same ages at which metal content was determined. Liver and spleen were extracted from saline-perfused animals to avoid blood protein contamination. After extraction, the dissected structures were immediately frozen in liquid nitrogen and stored at −80°C. Samples were homogenized and briefly sonicated in lysis buffer (50 mM Tris-HCl pH 7.5, 150 mM NaCl, 1 mM EDTA, 0.5% CHAPS, 10% glycerol, proteases inhibitors cocktail). Debris were removed by centrifugation (10 min, 2000 × g, 4°C) and protein concentration was determined by BCA assay. For each sample, the same protein amount (either 30 or 50 μg, depending on the protein) was separated on SDS–PAGE. On each gel, four *Prnp*^+/+^, and four *Prnp*^0/0^ samples of the same age, but deriving from different broods, were loaded, in order to compare them. The acrylamide concentration was chosen depending on the protein molecular weight. Proteins were transferred on nitrocellulose and, after 1 h in blocking solution, membranes were incubated overnight at 4°C with the primary antibody, except anti-β-Tubulin III, anti-β-Actin, anti-PrP, anti-Atp7b and anti-Fp1, which were incubated 1 h at RT. After incubation with the secondary antibody, membranes were developed with ECL detection reagent and recorded by the digital imaging system Alliance 4.7 (UVITEC, Cambridge, UK). Bands quantification was performed with Uviband 15.0 software (UVITEC). Each protein signal was normalized against either β-Actin or β-III Tubulin, depending on the protein molecular weight, the signal intensity and the secondary antibody. The normalized values were compared between *Prnp*^+/+^ and four *Prnp*^0/0^ samples by performing the Student's *T*-test analysis, setting 2-tailed distribution and unequal variance.

### Antibodies

The following primary antibodies were used in TBST with 5% milk: anti-Ctr1 1:500; anti-Sod1 1:1000; monoclonal anti-PrP SHA31 1 μg/ml; anti-PrP D18 1 μg/ml; anti-TfR1 1:500; anti-β-Tubulin III 1:5000; anti-β-Actin Peroxidase 1:10000; anti-Steap3 1:3000; anti-Atp7a 1:2000; anti-Ccs 1:400. The following primary antibodies were used in TBST with 5% BSA: anti-Cp 1:250; anti-Tf 1:2000; anti-human ferritin 1:1000; anti-FtH 1:200. The anti-Fp1 was used 1:1000 in TBST with 3% BSA and 5% milk. The anti-Atp7b N-WNPD#1 was incubated 1:4000 in PBST with 2% BSA, after blocking overnight at 4°C in PBS with 3% BSA, according to Prof. Lutsenko instruction.

### Oxidase activity

Oxidase activity was measured in different samples from *Prnp*^+/+^ to *Prnp*^0/0^. Spleen and liver were extracted from saline-perfused male mice (see *Metal Ions Measurements*) and homogenized in PBS with 1.5% Triton X-100 and protease inhibitors (Chen et al., 2004): entire spleen samples were homogenized in 1.5 mL, while a liver piece was weighted and homogenized in 6 volumes of buffer. Serum samples were collected as described in *Metal Ion Measurements* paragraph. All samples were either immediately processed for the enzymatic assay or stored at 4°C for 1–2 days. For each analyzed age, samples were collected from *Prnp*^+/+^ to *Prnp*^0/0^ mice at the same time to avoid biases due to different storage and ensuing different loss of enzymatic activity. Serum Cp activity was measured following the protocol published by Schosinsky (Schosinsky et al., 1974). Briefly, 50 μL serum (50 μL ddH_2_O for the blank) were added to 750 μL acetate buffer (100 mM sodium acetate, pH 5) and heated for 5 min at 30°C. *O*-dianisidine dihydrochloride was dissolved 7.88 mM in ddH_2_O, protected from the light, heated at 30°C for 5 min and added to the samples (200 μL per tube). The reaction was run for 2.5 h and stopped adding 2 mL of 9 M sulfuric acid, then the absorbance (Abs) peak at 540 nm was recorded. For spleen and liver, 100 μL of homogenate were added to 700 μL acetate buffer. After adding 200 μL substrate, the reaction was carried out for 6 h (necessary to get a detectable signal form tissues) and stopped with 2 mL 9 M sulfuric acid to obtain a clearly detectable peak. The enzymatic activity was expressed in International Units, in terms of substrate consumed: Concentration of substrate oxidized = (Abs × 3 × V)/(9.6 × T) μmol/mL per minute, where: 9.6 is the molar extinction coefficient of chromophore (ml μmol^−1^ cm^−1^); 3 = dilution factor for the final measured solution volume, taking into account the addition of 2 mL sulfuric acid to 1 mL of reaction; V = correction for sample volume used (50 μL for serum and 100 μL for spleen and liver in 1 mL final volume); T is the incubation time (min; Schosinsky et al., 1974).

To confirm the Cp contribution when measuring oxidase activity, different conditions were used. Before adding *O*-dianisidine, serum was pre-incubated at 30°C for 5 min with either 10 μM EDTA or 28 mM sodium azide or 200 μM cuprizone (Levine and Peisach, 1963; Curzon, 1966; Benetti et al., 2010), or pre-heated at 85°C for 5 min (Schosinsky et al., 1974). Afterwards, reactions and enzymatic activity were performed and calculated as described.

### Hepdicin determination in serum

Serum samples were collected as described in *Metal Ion Measurements* paragraph and stored at −80°C upon addition of proteases inhibitors cocktail. Samples were obtained from P15, P30, P90, P180, and P300 male *Prnp*^+/+^ and *Prnp*^0/0^ mice. Hepdicin levels were determined by using the ELISA kit for Hepdicin SEB979Mu following the manifacturer's instructions

### Statistics

All the experiments were carried out by using, at least, four biological replicates (i.e., 4 *Prnp*^+/+^ samples and 4 *Prnp*^0/0^). The obtained data were statistically analyzed by performing the Student's *T*-test, setting parameters corresponding to the unequal sample variants and two-tailed distribution. *P* < 0.05 were considered statistically significant.

## Results

### PrP^C^-null mouse serum contains lower levels of copper bound to Cp, oxidase activity and iron

**Figure 1:**
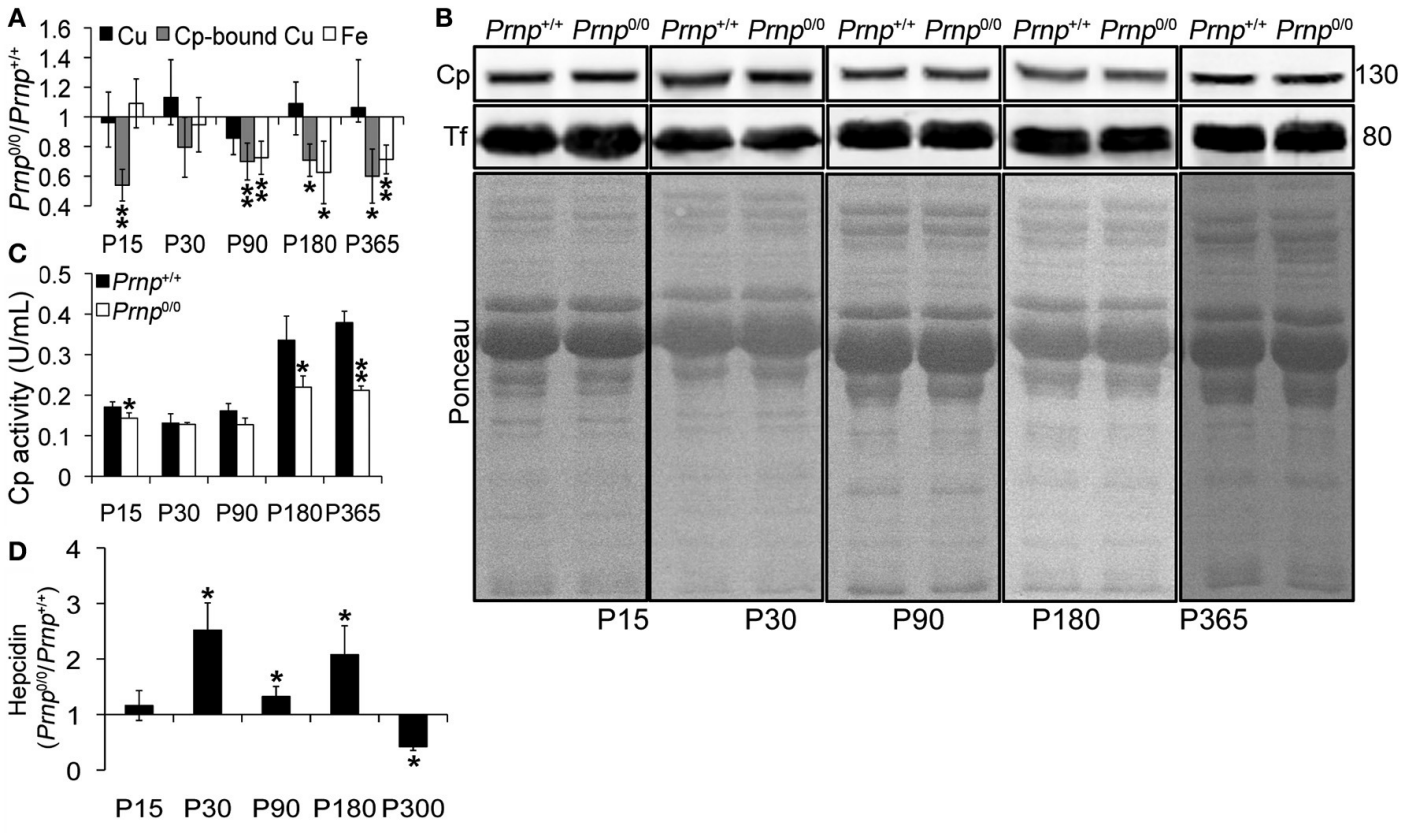
**Comparison of Cu, Cp-bound Cu, Fe, Cp and Tf expression, Cp activity, and Hepcidin levels in wild-type and PrP^C^-null mouse serum at different ages. (A)** The graph shows the ratio of Cu, Cp-bound Cu, and Fe levels in *Prnp*^0/0^ and *Prnp*^+/+^ serum samples (P15, P30, P180 *N* = 5; P90 *N* = 6; P365 *N* = 4). **(B)** Representative Western blot images showing Cp and Tf levels in *Prnp*^0/0^ and *Prnp*^+/+^ serum samples are shown. The equal protein loading is also shown by the ponceau staining. *N* = 4. **(C)** The graph shows the levels of Cp activity as U/mL in *Prnp*^0/0^ and *Prnp*^+/+^ serum (P15, P30, P180 *N* = 4; P90, P365 *N* = 3). **(D)** The graph shows the ratio of Hepcidin levels in *Prnp*^0/0^ and *Prnp*^+/+^ serum samples (*N* = 4). All error bars indicate SD; ^*^p < 0.05; ^**^p < 0.01.

To study the role of PrP^C^ in metal metabolism, we first measured copper and iron content in wild-type and PrP^C^-null mouse blood serum. We performed this analysis at different ages, from P15 to 1-year-old, and expressed results as the ratio between *Prnp*^0/0^ and *Prnp*^+/+^ ion concentration values. Results expressed in μg/mL are reported in Figures S1A–C. We found that the total amount of serum copper is not affected by PrP^C^ absence, as shown in Figure 1A. In physiological conditions, most of serum copper (~95%) is bound and transported by Cp (Harris and Gitlin, 1996). The remaining fraction is bound to macroglobulins and albumin, and represents the so-called copper exchangeable pool (Favier and Ruffieux, 1988; Linder and Hazegh-Azam, 1996). By dyalizing serum against the divalent cation chelator EDTA, we determined copper bound to Cp. Indeed, EDTA affinity constant for copper is enough for chelating free and exchangeable copper ions in serum (e.g., copper ions bound to albumin or small molecules) but it is unable to complex Cp-bound copper (Favier and Ruffieux, 1988). At all ages, Cp-bound copper is significantly decreased in PrP^C^-null mouse serum compared to wild-type animals (Figure 1A), suggesting a larger copper exchangeable pool fraction in PrP^C^-null mice. The reduced amount of Cp-bound copper in PrP^C^-null mouse serum seems not due to differences in Cp expression level, as shown in Figure 1B, and suggests an impairment in Cp ferroxidase activity. Measuring serum Cp activity, we confirmed that it is lessened in *Prnp*^0/0^ mice compared to wild-type (Figure 1C). To verify the reduced Cp activity in PrP^C^-null mice, Cp activity was determined in the presence of EDTA as divalent cations chelator (e.g., iron, copper), cuprizone as selective copper chelator unable to sequester copper in the ceruloplasmin catalytic site, azide as specific inhibitor of type 3 copper center responsible for ceruloplasmin activity, and by heating samples for denaturating ceruloplasmin so preventing its activity (Figure S2). While a slightly reduction in oxidase activity of both wild type and PrP-null mice in the presence of EDTA was observed, oxidase activity was strongly inhibited by adding the specific inhibitor azide and by heating serum, confirming the role of Cp in mediating oxidase activity. No alterations in oxidase activity were observed adding CZ. Since Cp ferroxidase activity mediates Fe^2+^ oxidation to Fe^3+^ for incorportation into transferrin (Tf), which is the main iron transporter, and iron mobilization from tissues (Osaki et al., 1966, 1971), we measured iron content in PrP^*C*^-null and wild-type mouse sera. Starting from P90, serum iron concentration was reduced in *Prnp*^0/0^ mice compared to wild-type (Figure 1A). However, no differences in serum Tf levels were observed (Figure 1B).

In PrP^C^-null mouse serum, the lower level of copper-loaded Cp (holoCp) results in lower oxidase activity, reduced iron export from tissues and ensuing serum iron content. Being the total amount of copper and Cp not altered, the mechanism of copper loading into Cp is most likely the impaired pathway in *Prnp*^0/0^ mice.

Beside copper loading into Cp, another pathway altered in *Prnp*^0/0^ mice is the iron uptake and/or transport from the duodenum to the blood stream (Singh et al., 2009b), and together the alterations likely cause serum iron deficiency. These observations indicate that PrP^C^ determines both Cp functionality and iron uptake/transport from duodenum.

### PrP^C^-null mouse liver shows evidence of iron accumulation and deficit in oxidase activity, leading to hepcidin production

**Figure 2:**
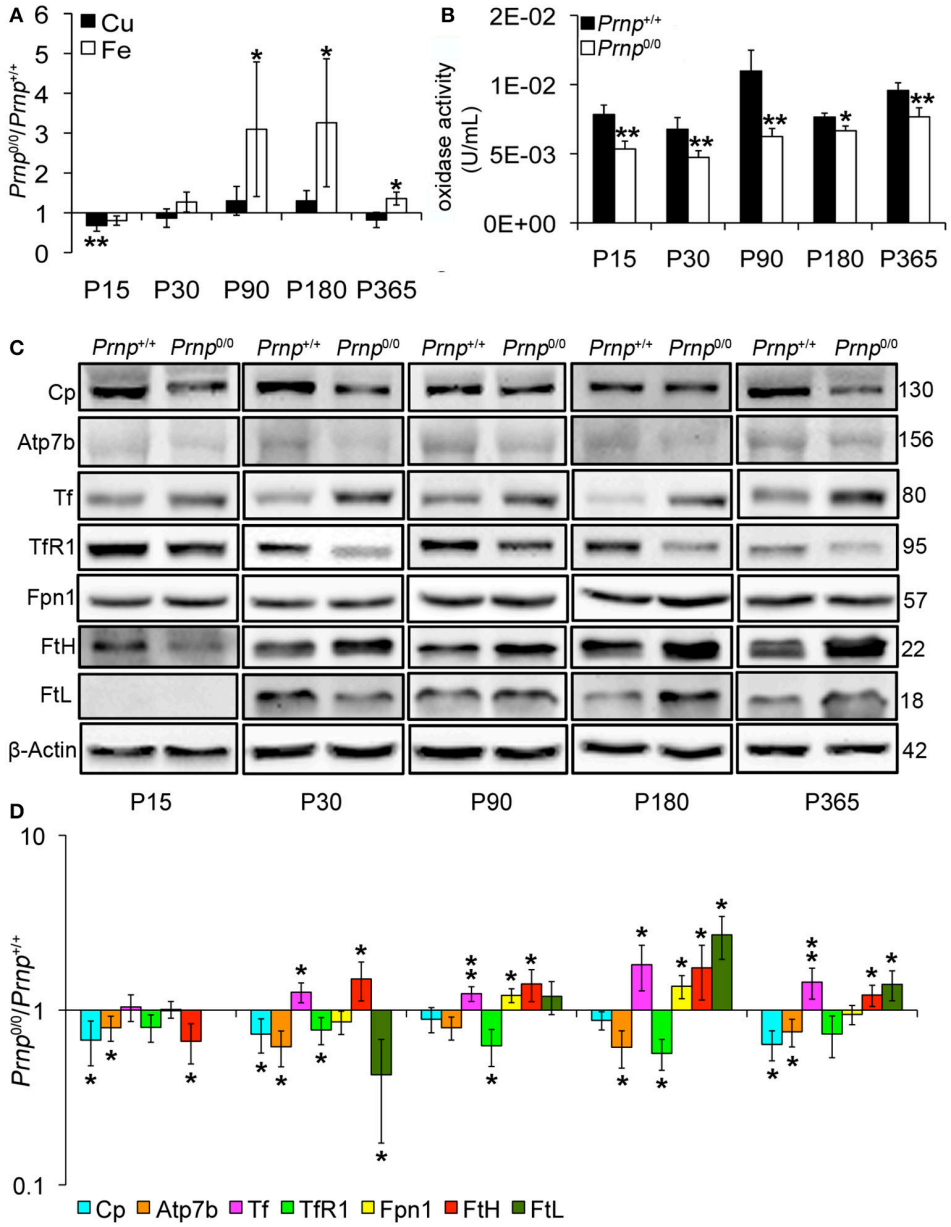
**Comparison of Cu, Fe, oxidase activity, metal-binding protein expression levels in wild-type and PrP^C^-null mouse liver at different ages. (A)** The graph shows the ratio of Cu and Fe levels in *Prnp*^0/0^ and *Prnp*^+/+^ liver samples (P15, P90 *N* = 5; P30, P180, P365 *N* = 4). **(B)** The graph shows the levels of oxidase activity as U/mL in *Prnp*^0/0^ and *Prnp*^+/+^ liver (P15, P90, P180, P365 *N* = 4; P30 *N* = 5). **(C)** Representative Western blot images showing metal-binding protein levels in *Prnp*^0/0^ and *Prnp*^+/+^ liver samples. The constant level of the housekeeping protein (β-Actin) are also reported. **(D)** The graph shows the up- or down-regulation of protein expression in *Prnp*^0/0^ samples compared to *Prnp*^+/+^, i.e., (*Prnp*^0/0^ protein OD/housekeeping OD)/(*Prnp*^+/+^ protein OD/housekeeping OD), *N* = 4. All error bars indicate SD; ^*^p < 0.05; ^**^p < 0.01.

Incorporation of copper into Cp mainly occurs in the liver (Terada et al., 1995). Therefore, we measured copper content in *Prnp*^0/0^ and *Prnp*^+/+^ mouse liver during early development and adulthood, and expressed results as the ratio between *Prnp*^0/0^ and *Prnp*^+/+^ ion concentration values (Figure 2A). Results expressed in μg/mL are reported in Figures S1D,E. Similarly to serum, total copper content is not overall altered in *Prnp*^0/0^ mouse liver, though a decrease of about 30% occurs at the early stage P15 (Figure 2A). Our data show iron accumulation in the liver starting from P90 (Figure 2A), corresponding to the reduction of serum iron concentration (Figure 1A). As for serum Cp activity, liver oxidase activity is diminished in *Prnp*^0/0^ mice (Figure 2B). The activity of ferroxidases is necessary for iron efflux from stores via Ferroportin1 (Fpn1). Indeed, in absence of ceruloplasmin activity, iron accumulates in the liver causing anemia (Osaki et al., 1971; Harris et al., 1999; Kosman, 2010; Ganz, 2013). Therefore, the decreased oxidase activity we observed in *Prnp*^0/0^ mouse liver and serum is likely the cause of iron accumulation in the liver and decrease in the serum.

Since contrasting data on PrP^C^ expression in mouse liver are reported in literature (Miele et al., 2003; Peralta and Eyestone, 2009; Peralta et al., 2012; Arora et al., 2015), and considering that 50 μg of liver protein extract were not enough to detect a clear PrP^C^ signal (data not shown), we verified PrP^C^ expression by performing immunoprecipitation from *Prnp*^+/+^ mouse liver, with *Prnp*^0/0^ as negative control. The result confirmed the expression of PrP^C^ in this organ (Figure S3).

Serum iron homeostasis is regulated by a small peptide hormone, hepcidin, produced by hepatocytes (Ganz, 2013). Hepcidin binds divalent metal transporter 1 and Fpn1 and induces their endocytosis and degradation. In this way, it prevents iron absorption from enterocytes, and iron release from iron stores and (mainly splenic) macrophages recycling senescent erythrocytes. Hepcidin synthesis is regulated by iron levels in intracellular stores and blood (Aoki et al., 2005; Detivaud et al., 2005; Gehrke et al., 2005; Ramos et al., 2011; Feng et al., 2012). Upon iron accumulation hepcidin expression is induced, while it is reduced when hepatocyte and/or blood iron content decreases. Measuring *Prnp*^0/0^ and *Prnp*^+/+^ mouse serum hepcidin levels, we found higher hepcidin content in PrP^C^-null mice from P30 to P180, lowering at P300 compared to wild-type mice (Figure 1D). Higher hepcidin levels in adult *Prnp*^0/0^ mice is in agreement with liver oxidase activity impairment and iron overload. In 1-year-old *Prnp*^0/0^ mice, hepcidin is decreased and hepatic iron content reduced. Hepcidin reduction is likely due to the prolonged serum iron deficiency (Pigeon et al., 2001; Nicolas et al., 2002).

Focusing on copper and iron metabolic pathways in the liver, we found that Cp and copper-transporting ATPase 2 (Atp7b) expression levels are lower in *Prnp*^0/0^ mice compared to wild-type animals (Figures 2C,D). These data, combined with lower serum holoCp level and activity, suggest the formation of apoCp and its intracellular degradation within hepatocytes (Hellman and Gitlin, 2002). To exclude alterations at trascriptional level, we compared *Prnp*^0/0^ and *Prnp*^+/+^ mice GPI anchored Cp mRNA levels by qRT-PCR. No differences in Cp transcription were observed (Figure S4A), suggesting that Cp decrease is caused by a higher degradation rate. These findings support the reduced Atp7b levels in PrP-null mice. Indeed, Atp7b is devoted both to excrete copper into the bile and translocate it to the *trans*-Golgi network (TGN) for its incorporation in copper-binding proteins such as Cp (Gupta and Lutsenko, 2009). Lower Atp7b expression levels in *Prnp*^0/0^ mice may result in failure of copper incorporation into Cp, production of apo-Cp, and rapid degradation.

Iron metabolic pathway is modulated by intracellular iron content *via* iron-responsive proteins and iron-responsive elements interaction. Intracellular iron accumulation reduces iron uptake molecular mechanisms, like transferrin receptor 1 (TfR1), increasing iron export and storage mechanisms such as Fpn1, ferritin H (FtH) and ferritin L (FtL) (Zecca et al., 2004; Anderson et al., 2007). In *Prnp*^0/0^ liver, the expression level of iron importer TfR1 is reduced, while iron exporter Fpn1 follows iron accumulation trend reaching the highest expression level at ages P90-P180, the period with the highest iron content (Figures 2C,D). Tf level is also increased in PrP^C^-null liver compared to wild-type liver. Similarly to the other iron-binding proteins, storage proteins FtH and FtL are differently expressed in *Prnp*^0/0^ liver. In particular, FtH was less expressed at P15, when also iron is slightly lower (though not statistically significant). Then, FtH starts being overexpressed in agreement with iron accumulation. FtL tightly follows iron levels since it is not expressed in P15 liver, is downregulated at P30, but overexpressed in aged mice in agreement with iron overload (Figures 2C,D). In 1-year-old PrP-null liver, its expression decreases in agreement with iron content reduction. The different modulation of FtH and FtL expression in *Prnp*^0/0^ liver can be related to a different regulation at post-transcriptional level. Indeed, FtH and FtL independently respond to iron levels, and FtL is more tightly regulated by iron at a post-transcriptional level than FtH (Sammarco et al., 2008; Arosio et al., 2009).

Iron accumulation is not due to changes in six-transmembrane epithelial antigen of prostate 3 (Steap3). This protein is highly expressed in the liver and acts as metalloreductase in iron and copper uptake (Gomes et al., 2012). Conversely from Cp, the expression level of Steap3 is not altered in the liver of *Prnp*^0/0^ mice (Figure S4B).

Overall, these data suggest that PrP^C^ absence does not alter the global copper content but impairs the pathway of copper loading into Cp. Consequently, Cp activity is compromised, and iron is accumulated in the liver and not delivered to serum. Although we observe the same modulation in Tf expression, our data concerning liver iron content and Ferritin expression are not in agreement with what previously published by Singh and colleagues (Singh et al., 2009b), i.e., lower iron, FtH and FtL levels in PrP^C^-null liver. Despite these discrepancies, our data confirm iron dyshomeostasis in PrP^C^-null mice and support the diminished oxidase activity in both liver and serum of *Prnp*^0/0^ mice as the cause of serum iron deficiency.

### PrP^C^-null mouse spleen shows decreased copper content and iron accumulation which causes splenomegaly

**Figure 3:**
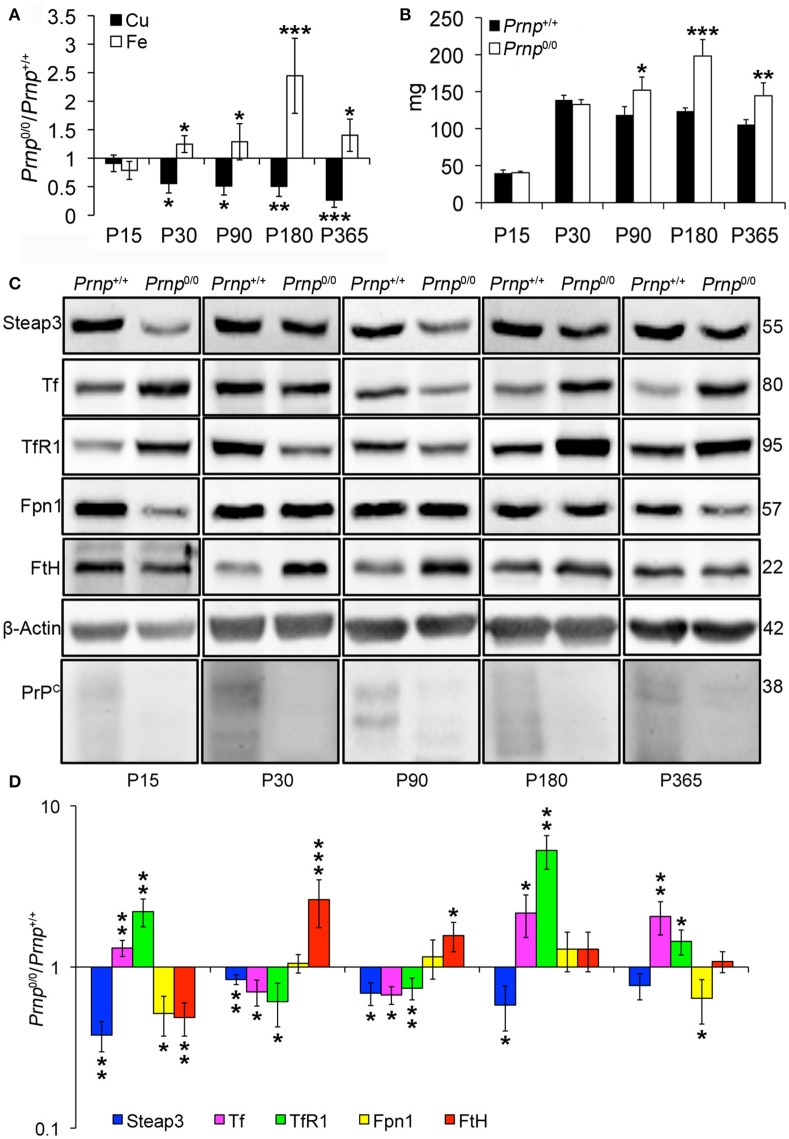
**Comparison of Cu, Fe, and metal-binding protein expression levels in wild-type and PrP^C^-null mouse spleen at different ages. (A)** The graph shows the ratio of Cu and Fe levels in *Prnp*^0/0^ and *Prnp*^+/+^ spleen samples (P15 *N* = 3; P30 *N* = 4; P90, P180 *N* = 6; P365 *N* = 5). **(B)** The graph shows the weight of spleen extracted from *Prnp*^0/0^ and *Prnp*^+/+^ mice; *N* = 4. **(C)** Representative Western blot images showing metal-binding protein levels in *Prnp*^0/0^ and *Prnp*^+/+^ spleen samples. The constant level of the housekeeping protein (β-Actin) are also reported. **(D)** The graph shows the up- or down-regulation of protein expression in *Prnp*^0/0^ samples compared to *Prnp*^+/+^, i.e., (*Prnp*^0/0^ protein OD/housekeeping OD)/ (*Prnp*^+/+^ protein OD/housekeeping OD); *N* = 4. All error bars indicate SD; ^*^p < 0.05; ^**^p < 0.01; ^***^p < 0.001.

Taking into account the alterations observed in the liver of *Prnp*^0/0^ mice for iron and copper metabolism, we investigated the effects of PrP^C^ absence on spleen, another fundamental organ for metal ion homeostasis. First, we confirmed the presence of PrP^C^ in the spleen (Figure S3). Then, we measured copper and iron content in wild-type and PrP^C^-null mouse spleen at the different ages, and expressed results as the ratio between *Prnp*^0/0^ and *Prnp*^+/+^ ion concentration values (Figure 3A). Results expressed in μg/mL are reported in Figures S1F,G. Conversely from the liver, we detected a strong reduction in copper content and an increase in iron level starting from P30 in *Prnp*^0/0^ spleen (Figure 3A). The copper content reduction is likely related to alterations in its uptake due to PrP^C^ ablation and lower Steap3 expression detected in PrP^C^-null spleen (Figures 3C,D). Steap3 is indeed involved in reducing Cu^2+^ to Cu^+^ for subsequent internalization via Ctr1. Reduced Steap3 level suggests an impairment of copper uptake leading to copper deficiency in PrP^C^-null mouse spleen. Expression levels of the copper-binding proteins Cp and Atp7b revealed no differences between *Prnp*^+/+^ and *Prnp*^0/0^ mouse spleen (Figure S5A), suggesting the correct incorporation of copper into Cp. Indeed, oxidase activity in spleen is not altered in PrP^C^-null mice, as shown in Figure S5B.

Similarly to the liver, iron metabolic pathway is altered in *Prnp*^0/0^ mouse spleen earlier than iron accumulation (Figures 3C,D), with the upregulation of Tf and TfR1. The subsequent iron accumulation, occurring at P30 and P90 in *Prnp*^0/0^ splenocytes, leads to downregulation of TfR1 and Tf, and upregulation of FtH. At these ages, Fpn1 level is not affected by PrP^C^ absence, probably due to increased serum hepcidin responsible for its degradation. In adult PrP^C^-null mice TfR1 and Tf levels are upregulated, while Fpn1 expression level is reduced. Therefore, we can hypothesize that spleen iron overload can be linked to the drop in serum Cp activity, which is necessary for iron efflux from Fpn1, and to the expression levels of Tf, TfR1 and Fpn1. These changes lead to splenomegaly, an enlargement of the spleen caused by iron overload (Figure 3B). Splenomegaly is commonly related to anemia and copper deficiency (Guo et al., 2013; Shawki et al., 2015), as described by our results for PrP^C^-null mice in previous paragraphs.

The atypical response to iron overload is related to concomitant copper reduction in PrP^C^-null spleen. Indeed, iron and copper homeostasis share common proteins and their regulation is closely interconnected. For instance, the unexpected TfR1 overexpression in presence of iron accumulation is due to copper deficiency, as previously reported for copper deficient rats (Auclair et al., 2006; Andersen et al., 2007). The iron overload we observe in *Prnp*^0/0^ spleen is in contrast with findings previously published (Singh et al., 2009b), but spleen iron accumulation is consistent with our data showing serum iron deficiency and splenomegaly.

### PrP^C^-null mouse CNS balances copper and iron homeostasis dysregulation by modulating transporters expression

**Figure 4:**
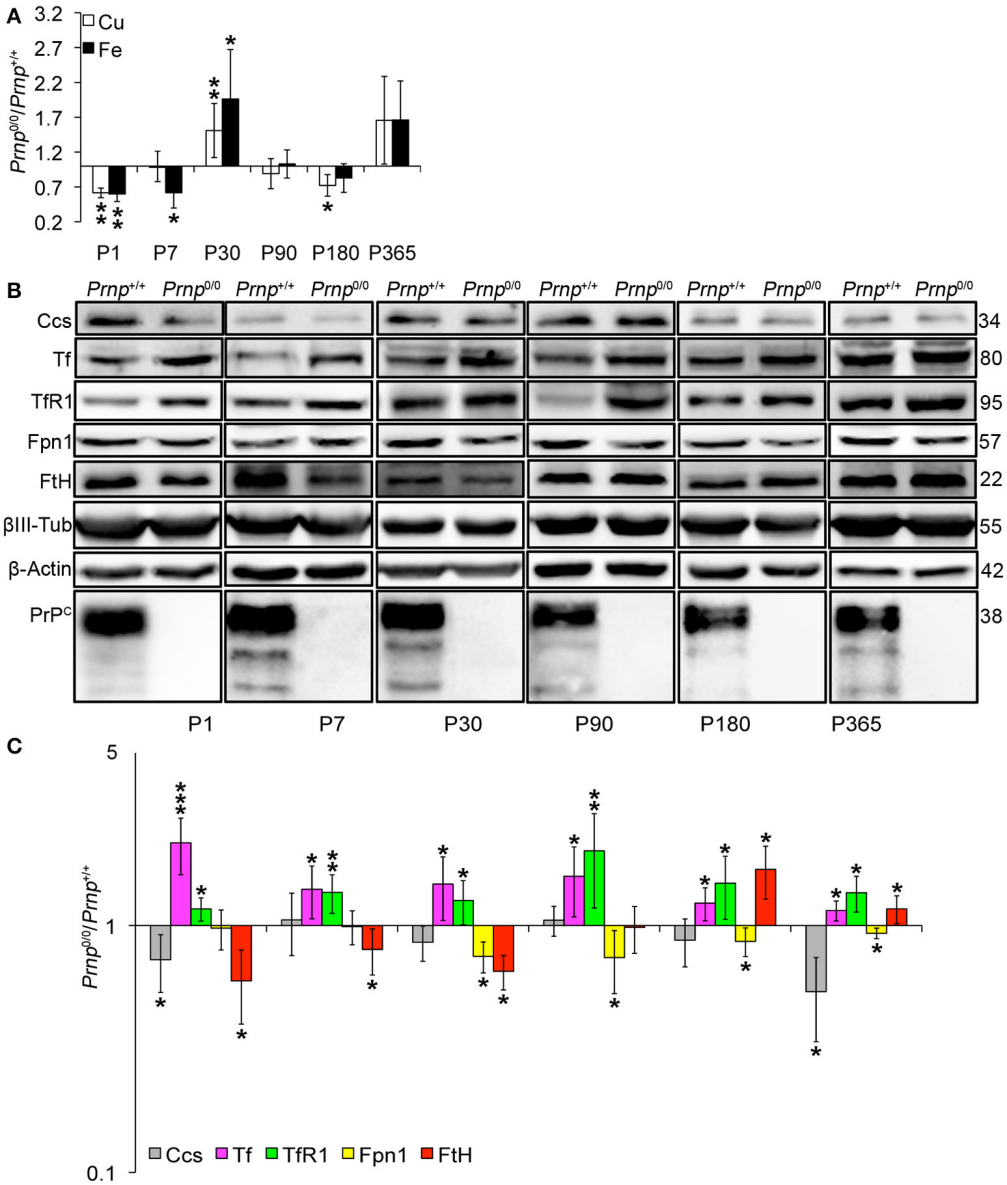
**Analysis of Cu and Fe content and metal-binding protein expression in wild-type and PrP^C^-null mouse total brain at different ages. (A)** The graph shows the ratio of Cu and Fe levels in *Prnp*^0/0^ and *Prnp*^+/+^ brain samples (P1, P365 *N* = 4; P7, P30, P180 *N* = 6; P90 *N* = 5). **(B)** Representative Western blot images showing metal-binding protein levels in *Prnp*^0/0^ and *Prnp*^+/+^ brain samples (*N* = 4). The constant level of the housekeeping proteins (β-III Tubulin and β-Actin) are also reported. **(C)** The graph shows the up- or down-regulation of protein expression in *Prnp*^0/0^ samples compared to *Prnp*^+/+^, i.e., (*Prnp*^0/0^ protein OD/housekeeping OD)/(*Prnp*^+/+^ protein OD/housekeeping OD). All error bars indicate SD; *N* = 4 minimum; ^*^p < 0.05; ^**^p < 0.01; ^***^p < 0.001.

**Figure 5:**
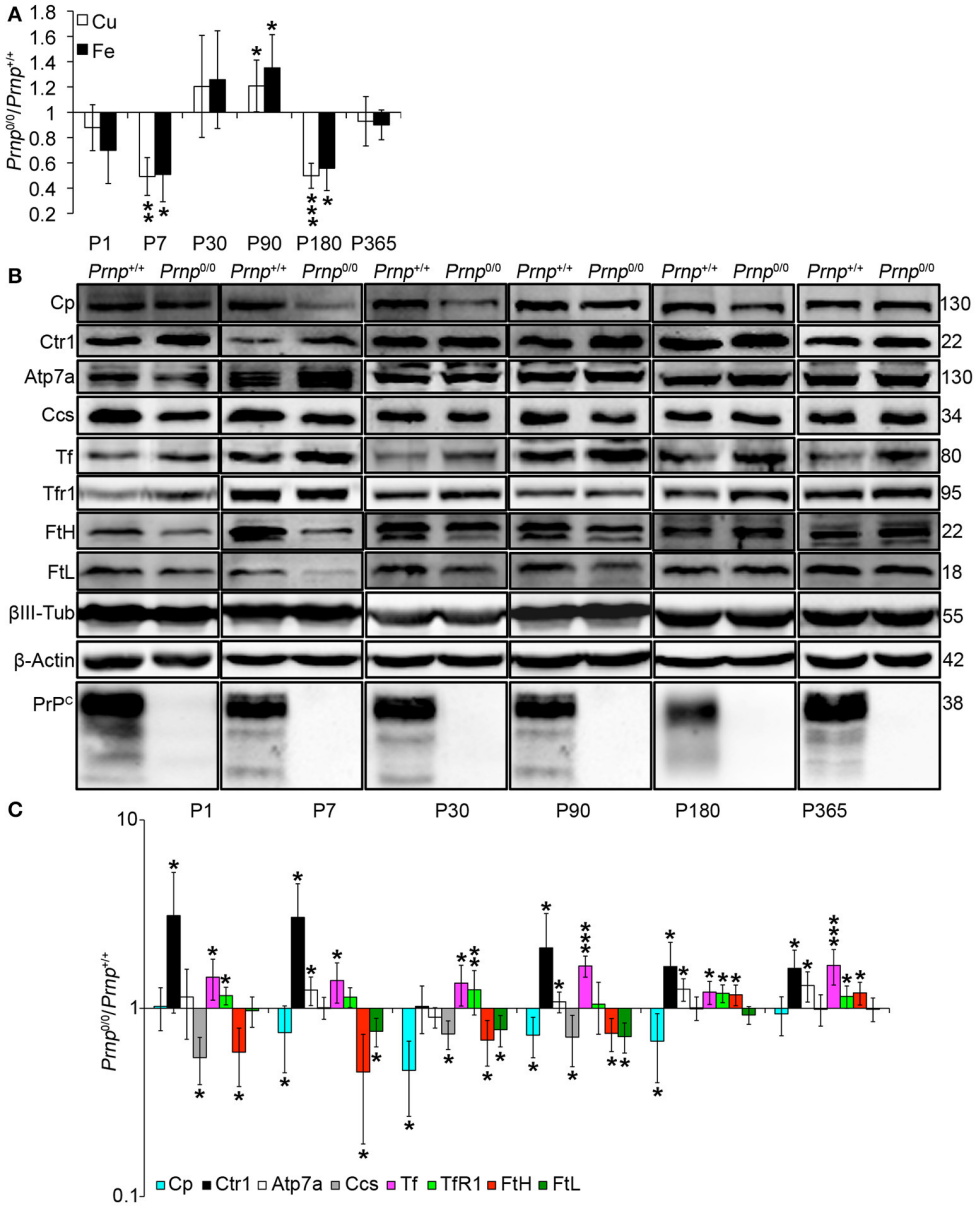
**Analysis of Cu and Fe content and metal-binding protein expression in wild-type and PrP^C^-null mouse isolated hippocampus at different ages. (A)** The graph shows the ratio of Cu and Fe levels in *Prnp*^0/0^ and *Prnp*^+/+^ hippocampus samples (P1, P180, P365 *N* = 4; P7 *N* = 7; P30 *N* = 6; P90 *N* = 5). **(B)** Representative Western blot images showing metal-binding protein levels in *Prnp*^0/0^ and *Prnp*^+/+^ hippocampal samples (*N* = 4). The constant level of the housekeeping proteins (β-III Tubulin and β-Actin) are also reported. **(C)** The graph shows the up- or down-regulation of protein expression in *Prnp*^0/0^ samples compared to *Prnp*^+/+^, i.e., (*Prnp*^0/0^ protein OD/housekeeping OD)/(*Prnp*^+/+^ protein OD/housekeeping OD). All error bars indicate SD; *N* = 4 minimum); ^*^p < 0.05; ^**^p < 0.01; ^***^p < 0.001.

Since CNS is the organ with the highest PrP^C^ expression (Figure S3) and primary target of prion disorders, we analyzed the impact of PrP^C^ absence on copper and iron metabolism. We analyzed the total brain and the isolated hippocampus. The latter is the region showing PrP^C^ expression at synapse and the most prominent alterations in *Prnp*^0/0^ mouse model. We first measured copper and iron content in wild-type and PrP^C^-null mouse total brain and isolated hippocampus at different ages, from P1 to 1-year-old, and expressed results as the ratio between *Prnp*^0/0^ and *Prnp*^+/+^ ion concentration values (Figures 4A, 5A). Results expressed in μg/mL are reported in Figures S1H–K. Both copper and iron show a fluctuating behavior along ages in both total brain and hippocampus, with a reduction in their content at early stages (P1 and P7) and adulthood (P180), and an increase at P30-P90 (Figures 4A, 5A).

By analyzing the expression pattern of copper and iron metabolism proteins, we found some modulations in *Prnp*^0/0^ mouse total brain and isolated hippocampus which, at the same time, can suggest a response to metal ion altered metabolism. Copper chaperone for Sod1 (Ccs) is the only copper-binding protein altered in total brain of PrP^C^-null mice (Figures 4B,C), while no differences were found for Ctr1, Atp7a, Atp7b, Steap3, Cp, and Sod1 (Figure S6). Iron metabolic pathway is highly affected in *Prnp*^0/0^ mouse total brain, as indicated by the higher levels of Tf and TfR1 and reduced expression of Fpn1 at all the considered ages (Figures 4B,C). FtH is downregulated in juvenile *Prnp*^0/0^ mice and strongly upregulated in the adulthood (Figures 4B,C), while no alterations in FtL level are found. The differential expression of Tf, TfR1 and Fpn1, as well as the FtH downregulation, correlates with iron deficiency phenotpe (Zecca et al., 2004).

In *Prnp*^0/0^ mouse hippocampus, proteins involved in both copper and iron metabolism are altered. The copper-binding protein Ctr1 and Atp7a are upregulated at all stages, while Cp and the intracellular chaperone Ccs are downregulated (Figures 5B,C). Ctr1 upregulation suggests a compensatory mechanism for copper uptake in the absence of PrP^C^. No changes are observed for Steap3, Atp7b, and Sod1 (Figure S7). Although Sod1 expression level is not affected by PrP^C^ absence, the downregulation of Ccs occurring in PrP^C^ knockout CNS may affect copper loading onto Sod1 (Culotta et al., 1997), leading to the formation of the apo-Sod1 form. Therefore, Ccs reduction may contribute to the diminished Sod1 antioxidant activity registered in PrP^C^-null mice (Brown and Besinger, 1998; Kralovicova et al., 2009). Concerning iron metabolism, TfR1 and Tf are increased along all ages, while the expression level of the iron-storage proteins FtH and FtL is decreased (Figures 5B,C). As observed in total brain, the altered expression of iron-binding proteins correlates with iron deficiency status (Zecca et al., 2004).

The presented results indicate that in the CNS of PrP^C^-null mice, the expression of copper- (Cp, Ctr1, Ccs1) and iron-binding proteins (Tf, TfR1, FtH, FtL, Fpn1) is regulated in line with a copper and iron deficiency status. These findings are in agreement with data previously reported (Singh et al., 2009b). The fluctuating alterations of both copper and iron content may result from the compensation induced by metal-binding protein expression modulation.

## Conclusions

Metal homeostasis is crucial to preserve physiological functions in living organisms, and alterations in their absorption, metabolism, and excretion have pathological implications. Neurodegenerative disorders are characterized by impairment of metal homeostasis, though the engaged molecular mechanisms are not well-understood. Through its multiple metal-binding sites, PrP^C^ interacts with metals and modulates their physiological functions, such as the S-nitrosylation of NMDA receptors (Gasperini et al., 2015). On the other hand, it has been shown that metals promote PrP aggregation (Jobling et al., 2001; Younan et al., 2011; Migliorini et al., 2014). The dual effect of metals on PrP depends on differences in their functional value. To understand the impact of PrP^C^ on metal homeostasis and their functional value, we investigated changes in copper and iron metabolism occurring in PrP^C^-null mice. The *Prnp* gene knockout mimicks PrP^C^ loss-of-function occurring in prion diseases. Therefore, our results could also extend our understanding of the molecular mechanisms underlying these pathologies.

**Figure 6:**
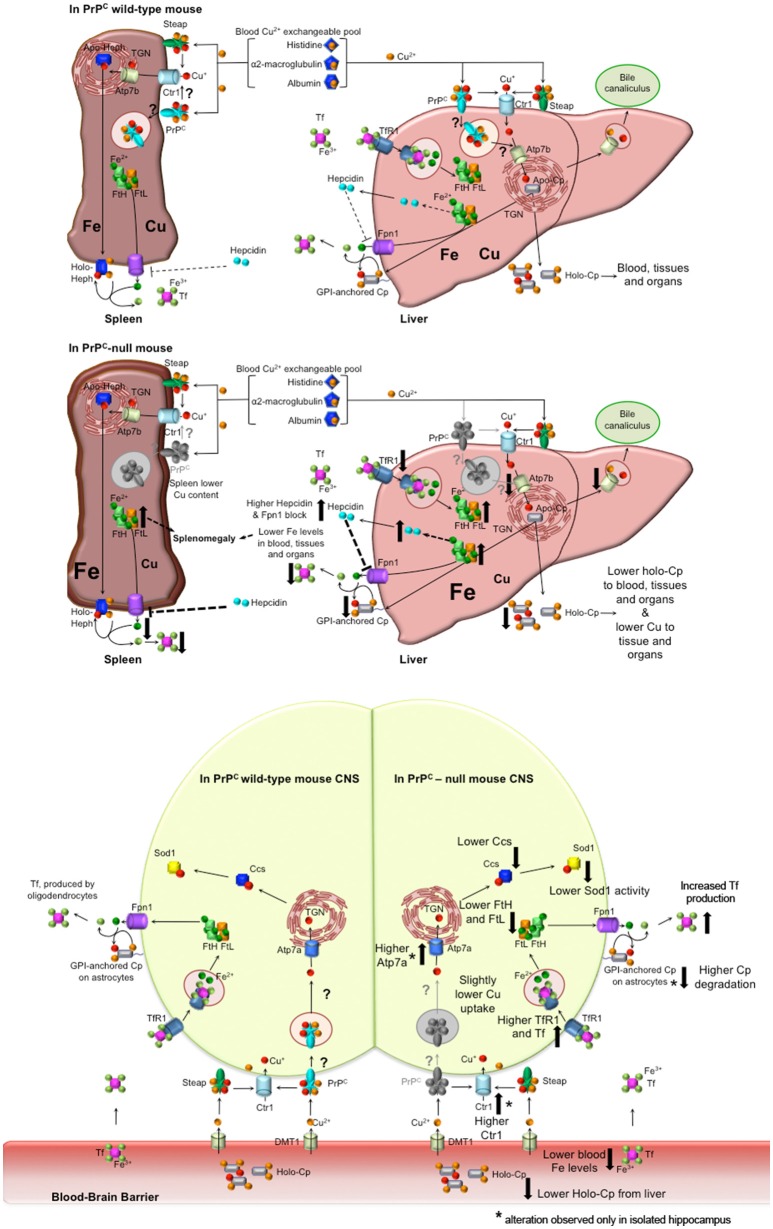
**Proposed model for PrP^C^ role in copper and iron homeostasis. Wild-type PrP^C^ mouse**. In both liver and spleen, PrP^C^ may bind Cu^2+^ released from the blood Cu^2+^ exchangeable pool, reduce to Cu^+^ and pass it to Ctr1 for its uptake, or may function itself as a transporter by internalization. Therefore, in the liver, PrP^C^ may be involved in the transport of copper to the TGN where Atp7b load it on Cp. Hence, holo-Cp can be released in the serum or GPI-anchored on hepatocytes, thus mediating iron export through its copper-dependent ferroxidase activity. **PrP^C^-null mice**. In the absence of PrP^C^, liver copper uptake is affected, hence the formation of holo-Cp is diminished. Consequently, iron is accumulated in the liver, TfR1 level is decreased, while FtH and FtL levels are increased. Therefore, hepcidin secretion is enhanced, with ensuing block of Fpn1. In the spleen, the absence of PrP^C^ induces a reduction in copper uptake that, together with hepcidin-mediated Fpn1 block, triggers iron accumulation, that, in turn, leads to splenomegaly. Liver and spleen iron accumulation results in decreased serum iron content. **Wild-type PrP^C^ CNS**. PrP^C^ may bind Cu^2+^ released from the blood Cu^2+^ exchangeable pool and reduce to Cu^+^, and pass it to Ctr1 for its uptake, or may function itself as a transporter by internalization. Therefore, PrP^C^ may be involved in the intracellular transport of copper, and loading on Ccs. **PrP^C^-null mouse CNS**. In PrP^C^-null blood, iron and Cp-bound copper levels are decreased. Therefore, compensatory mechanism maintain physiological copper and iron levels. In both total brain and isolated hippocampus, Tf, TfR1, FtH, FtL are modulated in response to iron deficiency. Copper-binding protein Ctr1, Atp7a and Cp are modulated in the hippocampus, responding to copper deficiency. Ccs is decreased in PrP^C^-null CNS, likely causing the decrease in Sod1 activity (Brown and Besinger, 1998; Kralovicova et al., 2009). All the alterations observed in PrP^C^-null mouse have been reported as big arrows pointing up in case of increase, pointing down in case of decrease. In the CNS scheme, ^*^flanking the arrow indicates an alteration observed only in the isolated hippocampus, but not in the total brain.

The results we obtained led us to hypothesize the model described in Figure 6. PrP^C^ absence causes a serum anemia with a subsequent accumulation in the liver and spleen. No differences in total copper content were observed in the serum and in the liver, but oxidase activity of the copper-dependent Cp was lower in PrP^C^-null mice compared to wild-type. The reduction in Cp activity is likely due to altered incorporation of copper into Cp with the subsequent formation of apo-Cp which is more prone to degradation (Hellman and Gitlin, 2002). The diminished oxidase activity affects Fe^2+^ to Fe^3+^ oxidation, thus impairing iron release from stores and its incorporation into Tf. The atypical behavior of iron and iron-binding proteins in the spleen may be due to the concurrent copper reduction in PrP^C^-null spleen (Auclair et al., 2006; Andersen et al., 2007).

Conversely from liver and spleen, copper and iron content in PrP^C^-null mouse CNS presents fluctuating concentrations implying the presence of homeostatic compensatory mechanisms. The brain has the highest metabolic rate of all organs and depends on oxidative metabolism for its energy. Therefore, it is not surprising that compensatory mechanisms are in place to avoid deficiency or dysmetabolism of essential metals, preventing adverse effects on CNS functions. Indeed, compensatory mechanisms maintain physiological levels of essential elements, in response to the altered iron and copper levels in PrP^C^-null mouse serum. This is exemplified by the modulation of TfR1,Tf, Ctr1, and Atp7a.

These results indicate that the absence of PrP^C^ leads to alterations in copper and iron functional values, with iron metabolism affected by low Cp activity. Interestingly, our data are in agreement with previously published works, showing a reduction in cerebrospinal fluid ferroxidase activity from Alzheimer's and Parkinson's disease patients (Boll et al., 1999, 2008; Capo et al., 2008; Olivieri et al., 2011). Similarly to our observations, they identified the impairment of copper incorporation in Cp as the main cause of ferroxidase activity reduction. The relevance of PrP^C^ loss-of-function on the modulation of copper metabolism has been confirmed in prion disorders. Indeed, the alteration of copper coordination by PrP^C^ is a key factor in prion pathologies, and can determine the disease onset (Stevens et al., 2009). Moreover, prion-infected brains show impairments in iron metabolism that resemble those observed in PrP^C^ knockout mice (Singh et al., 2009a). Our findings increase the understanding of PrP^C^ role in copper and iron metabolism, and provide a link with pathogenic mechanism involved in prion diseases.

## Author contributions

LG and EM performed the experiments and acquired the data. LG, EM, and FB analyzed the data and draft the work. FB designed the work. FB and GL interpreted the data for the work and critically revised the draft of the work. LG, EM, GL, and FB agreed to be accountable for all aspects of the work in ensuring that questions related to the accuracy or integrity or integrity of any part of the work are appropriately investigated and resolved.

### Conflict of interest statement

The authors declare that the research was conducted in the absence of any commercial or financial relationships that could be construed as a potential conflict of interest. The reviewer PC and handling Editor declared their shared affiliation, and the handling Editor states that the process nevertheless met the standards of a fair and objective review.
