# Supplementary material for: In Absence of the Cellular Prion Protein, Alterations in Copper Metabolism and Copper-Dependent Oxidase Activity Affect Iron Distribution
Source: Front Neurosci. 2016 Sep 27;10:437. doi: 10.3389/fnins.2016.00437 (PMC5037227; doi:10.3389/fnins.2016.00437)
Supplement: Supplementary file 1 [file DataSheet1.docx]

**Supplementary Information**

**IN ABSENCE OF THE CELLULAR PRION PROTEIN, ALTERATIONS IN COPPER METABOLISM AND COPPER-DEPENDENT OXIDASE ACTIVITY AFFECT IRON DISTRIBUTION**

Lisa Gasperini^1,2^, Elisa Meneghetti^1^, Giuseppe Legname^1,^* and Federico Benetti^1,3,^*

^1^Laboratory of Prion Biology, Department of Neuroscience, Scuola Internazionale Superiore di Studi Avanzati (SISSA), Trieste, Italy

^2^Current affiliation: Laboratory of Molecular and Cellular Neurobiology, Centre for Integrative Biology (CIBIO), Trento, Italy

^3^Current affiliation: ECSIN-European Center for the Sustainable Impact of Nanotechnology, ECAMRICERT SRL, Rovigo, Italy.

* To whom correspondence should be addressed:

- Federico Benetti, Viale Porta Adige 45, 45100 – Rovigo, Italy, phone + 39 0425 377501, e-mail [f.benetti@ecamricert.com](mailto:f.benetti@ecamricert.com)

- Giuseppe Legname, Via Bonomea 265, 34136 – Trieste, Italy, phone + 39 040 3787715, fax + 39 040 3787702, e-mail [legname@sissa.it](mailto:legname@sissa.it)

**1 Materials and Methods**

**1.1 RNA Extraction and qRT-PCR**

Ceruloplasmin (Cp) mRNA levels were analyzed by qRT-PCR in liver and hippocampal samples from post-natal day 15 (P15), P30 and P180 *Prnp*^+/+^ and *Prnp*^0/0^ male mice. Total RNA was extracted following the TRIzol reagent (15596, Invitrogen, Carlsbad, CA, USA) manufacturer's instructions. To eliminate genomic DNA contamination, samples were treated with RNase free DNase set (79254, Qiagen, Germantown, MD, USA) and purified using the RNeasy mini kit (74104, Qiagen). For each sample, 4 μg of RNA were retrotranscribed by using SuperScriptIII RT (18080, Invitrogen) and oligodT primer (5′-GCT GTC AAC GAT ACG CTA CGT AAC GGC ATG ACA GTG(T)_24_-3′). qRT-PCR was performed using iQ SYBR Green Supermix (170-8880, Bio-Rad, Hercules, USA) in an iCycler IQ Real Time PCR System (Bio-Rad). The initial amount of template of each sample was determined as relative expression versus housekeeping gene chosen as reference, i.e. glyceraldehyde 3-phosphate dehydrogenase (Gapdh) and β-Actin for liver samples, and Gapdh and βIII-Tubulin for hippocampus. The relative expression of each sample was calculated by the formula 2exp^-∆∆Ct^ (User Bulletin 2 of the ABI Prism 7700 Sequence Detection System) (Livak and Schmittgen, 2001). Housekeeping gene expression was not modified under the present experimental conditions. Non-template control and RT- control (i.e. retrotranscription reaction product obtained without the SuperScriptIII RT enzyme) were performed for each qRT-PCR reaction. mRNA of both secreted and GPI-anchored Cp were measured. Primer sequences for secreted Cp (sec_Cp), GPI-anchored Cp (GPI_Cp), β-Actin (βAct), βIII-Tubulin (βTub) and Gapdh were the following list: sec_Cp fwd TCCCTGGAACATACCAAACC, sec_Cp rev ATTTATTTCATTCAGCCAGACTTAG (Stasi et al., 2007); GPI_Cp fwd TCCCTGGAACATACCAAACC, GPI_Cp rev CCAGGTCATCCTGTAACTCTGA (Stasi et al., 2007); βAct fwd CACACCCGCCACCAGTTC, βAct rev CCCATTCCCACCATCACACC (Al-Sa'doni et al., 1997); βTub fwd CGCCTTTGGACACCTATTC, βTub rev TACTCCTCACGCACCTTG (Simonetti et al., 2008); Gapdh fwd TTCACCACCATGGAGAAGGC, Gapdh rev GGCATGGACTGTGGTCATGA (Chen et al., 2003).

**1.2 Immunoprecipitation**

Loading 30-50 μg of homogenate for Western blot was not enough to detect PrP^C^ expression in liver, while a not-well-defined signal was obtained in spleen. To assess whether PrP^C^ is expressed in these tissues or not, we performed an immunoprecipitation (IP). Brain, spleen and liver from *Prnp*^+/+^ and *Prnp*^0/0^ mice were homogenized and briefly sonicated in lysis buffer (50 mM Tris-HCl pH 7.5, 150 mM NaCl, 1 mM EDTA, 0.5% CHAPS, 10% glycerol, proteases inhibitors cocktail). Debris were removed by centrifugation (10 min, 2000 x g, 4 °C) and protein concentration was determined by BCA assay. The following quantities of protein extract were used in 1 mL of buffer volume: 1.3 mg of brain homogenate, 8.5 mg of liver homogenate, 5 mg of spleen homogenate. Samples were pre-cleared by 30 min incubation at 4 °C with protein G resin (17-0618-01 Amersham, UK). Then, the samples were incubated 2.5 h at 4 °C with 2 μg/mL of monoclonal anti-PrP SHA31 (A03213, BertinPharma, Montigny le Bretonneux, France), followed by 2 h incubation with protein G resin. Proteins eluted from the resin were processed for standard Western blot protocol and revealed with humanized Fab anti-PrP D18 (ABR-0D18, InPro Biotechnology, South San Francisco, CA, USA).**2 Supplementary Figures**


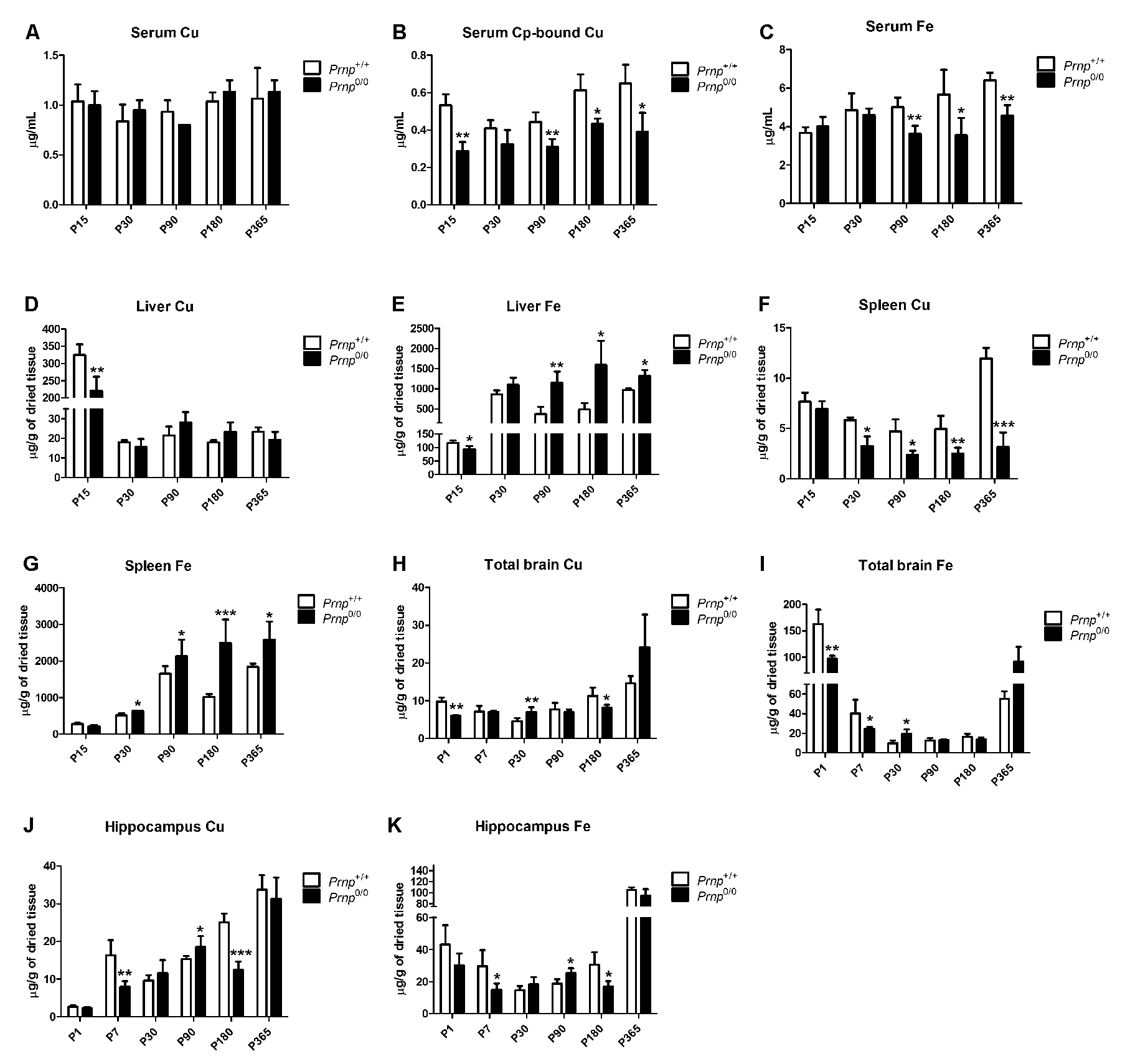


**Figure S1.**  The graphs report the measurements of copper and iron as μg/mL for serum and μg/g of dried tissue for liver and spleen. (**A**, **B**, **C**) Cu, Cp-bound Cu and Fe levels in *Prnp*^0/0^ and *Prnp*^+/+^ serum samples (P15, P30, P180 N=5; P90 N=6; P365 N=4). (**D**, **E**) Cu and Fe levels in *Prnp*^0/0^ and *Prnp*^+/+^ liver samples (P15, P90 N=5; P30, P180, P365 N=4). (**F**, **G**) Cu and Fe levels in *Prnp*^0/0^ and *Prnp*^+/+^ spleen samples (P15 N=3; P30 N=4; P90, P180 N=6; P365 N=5). (**H**, **I**) Cu and Fe levels in *Prnp*^0/0^ and *Prnp*^+/+^ total brain samples (P1, P365 N=4; P7, P30, P180 N=6; P90 N=5). (**J**, **K**) Cu and Fe levels in *Prnp*^0/0^ and *Prnp*^+/+^ hippocampus samples (P1, P180, P365 N=4; P7 N=7; P30 N=6; P90 N=5). All error bars indicate SD; **p*<0.05; ***p*<0.01; ****p*<0.001.


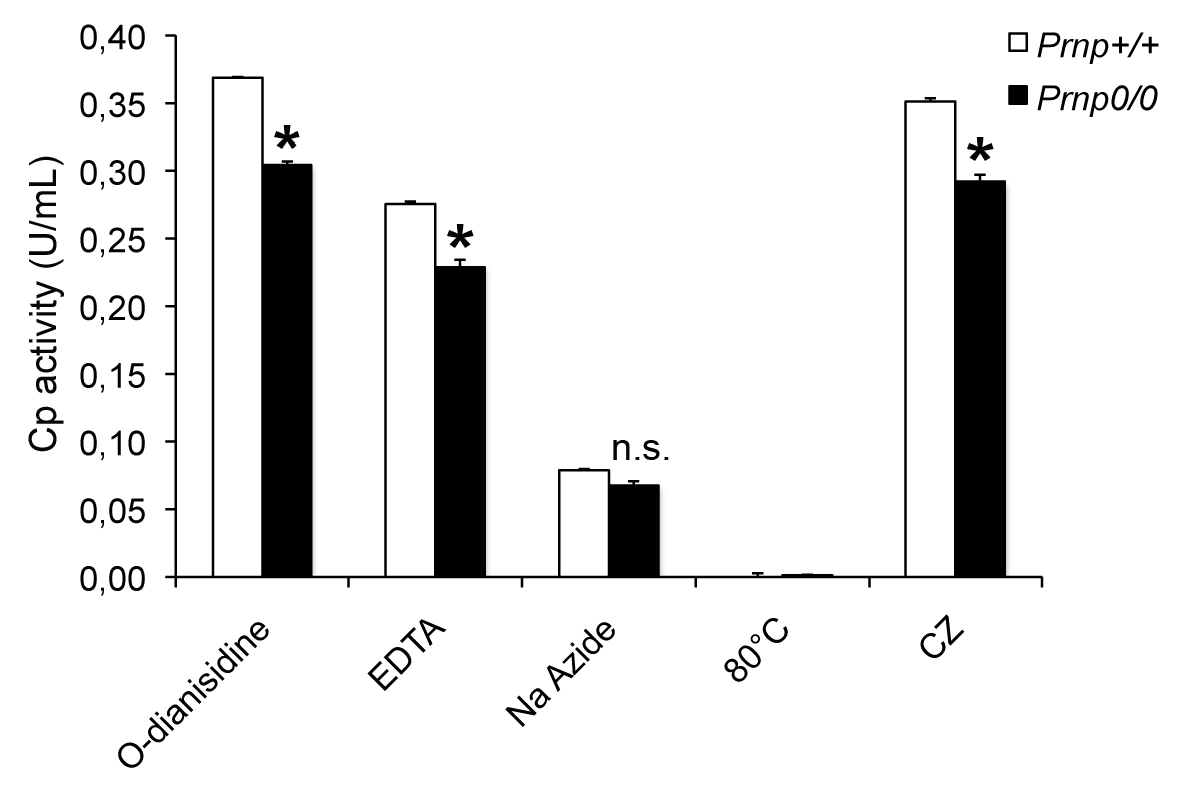


**Figure S2.**  The graph shows the levels of Cp activity as U/mL in adult *Prnp*^0/0^ and *Prnp*^+/+^ serum in different assay conditions: normal reaction with *O*-dianisidine, pre-incubation at 30 °C for 5 min with either 10 µM EDTA, or 28 mM sodium azide or 200 µM cuprizone (CZ) or at 80 °C for 5 min. N=3. All error bars indicate SD; **p*<0.05; n.s. = non significant.

**
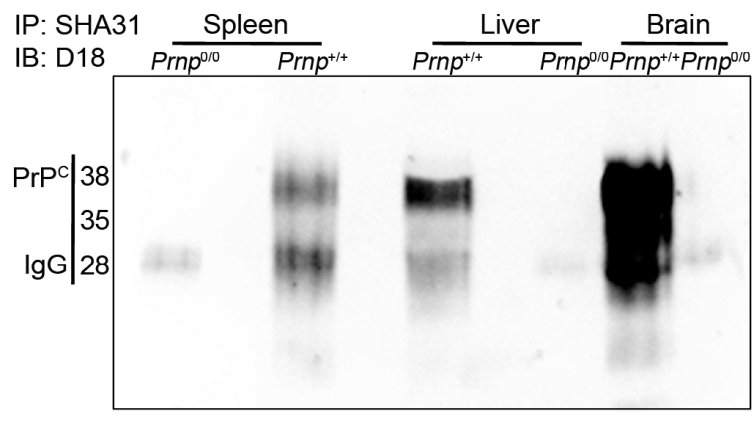
**

**Figure S3. Immunoprecipitation of PrP^C^ in mouse spleen, liver and brain.**

**
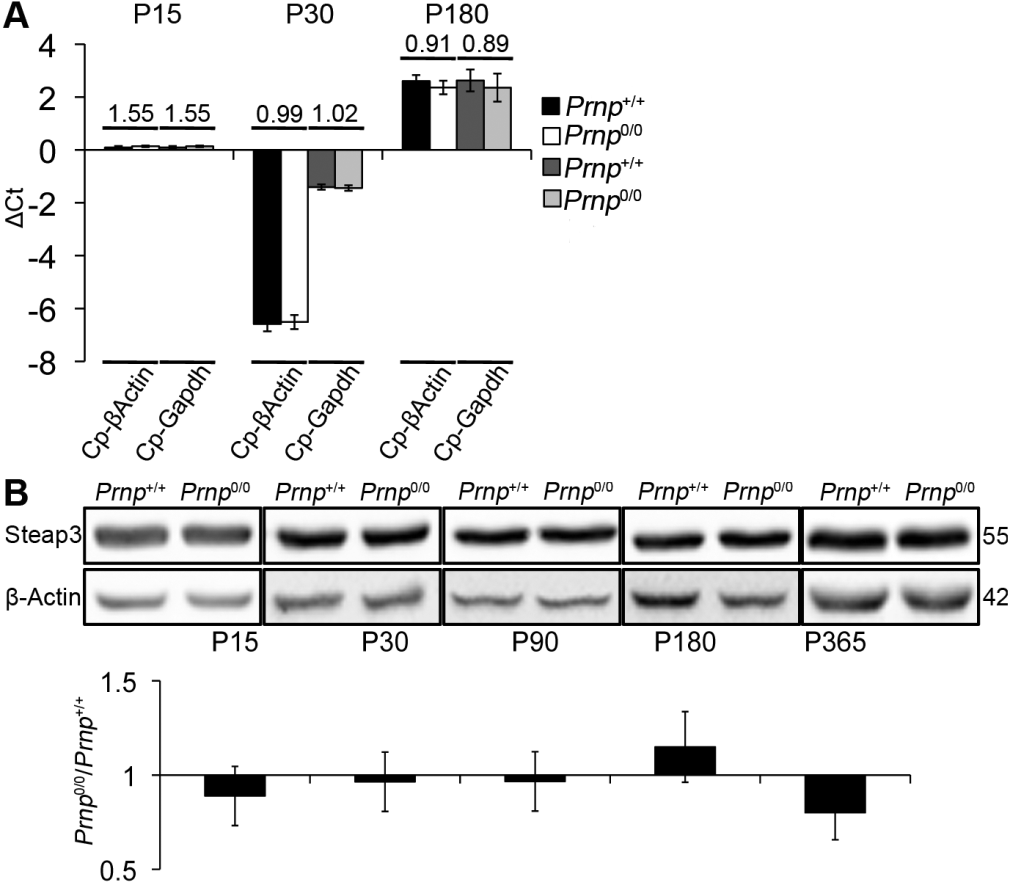
**

**Figure S4.**

(**A**) GPI anchored Ceruloplasmin (GPI-Cp) mRNA analysis by qRT-PCR in *Prnp*^0/0^ and *Prnp*^+/+^ liver at different ages (N=4). The graph shows the ΔCt values obtained by normalizing GPI-Cp Ct on two housekeeping genes (Gapdh and β-Actin). The numbers reported on the graph columns indicate the fold change values. (**B**) In the upper part of the panel, representative Western blot images showing Steap3 levels in *Prnp*^0/0^ and *Prnp*^+/+^ liver samples. The constant level of the housekeeping protein (β-Actin) are also reported. In the lower part of the panel, the graph shows the quantification of protein expression in *Prnp*^0/0^ samples compared to *Prnp*^+/+^, i.e. (*Prnp*^0/0^ protein OD/housekeeping OD)/ (*Prnp*^+/+^ protein OD/housekeeping OD). All error bars indicate SD, N=4.

**
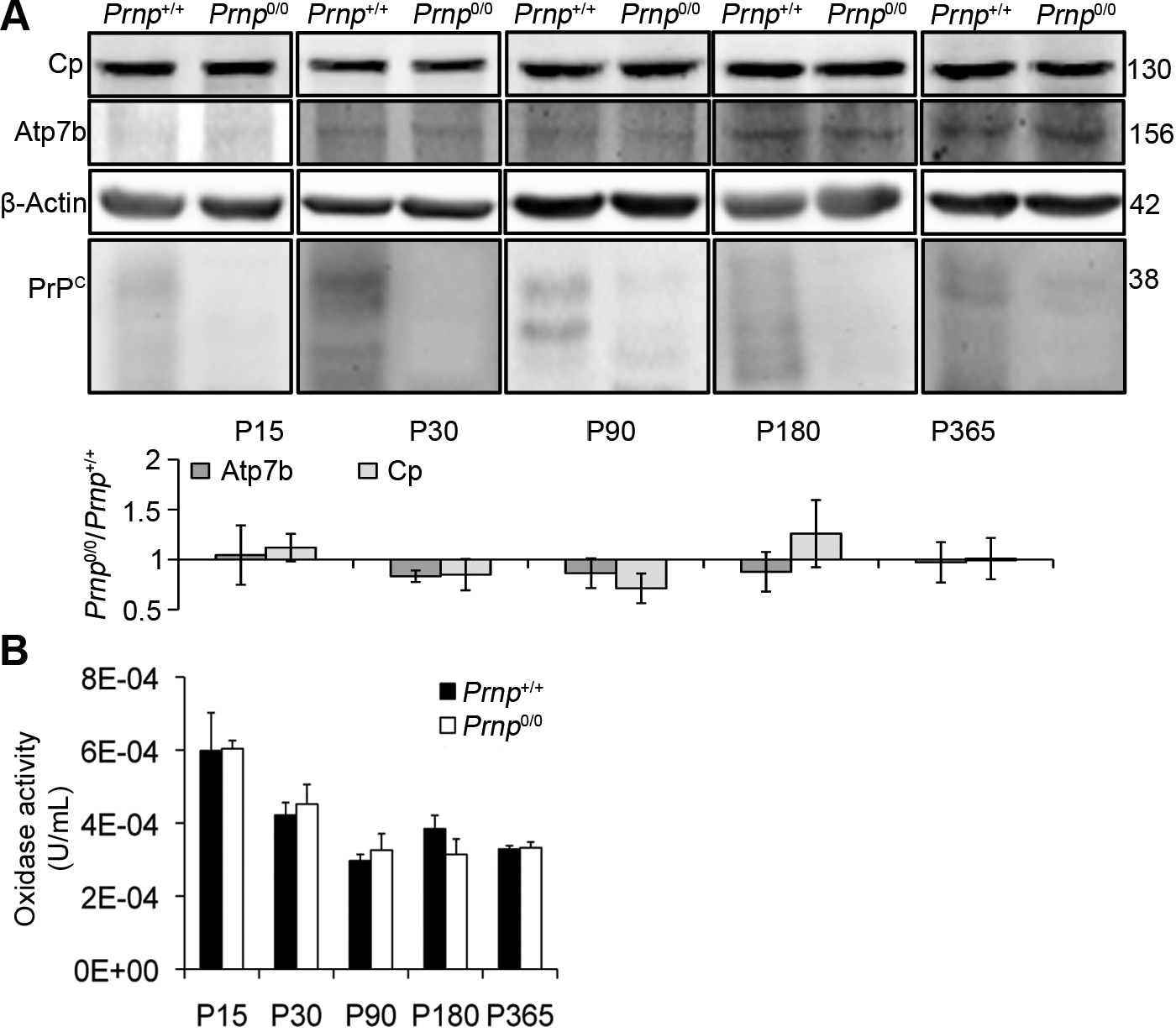
**

**Figure S5.**

(**A**) In the upper part of the panel, representative Western blot images showing Cp and Atp7b levels in *Prnp*^0/0^ and *Prnp*^+/+^ spleen samples. The constant level of the housekeeping protein (β-Actin) are also reported. In the lower part of the panel, the graph shows the quantification of protein expression in *Prnp*^0/0^ samples compared to *Prnp*^+/+^, i.e. (*Prnp*^0/0^ protein OD/housekeeping OD)/ (*Prnp*^+/+^ protein OD/housekeeping OD). All error bars indicate SD, N=4. (**B**) The graph shows the levels of oxidase activity as U/mL in *Prnp*^0/0^ and *Prnp*^+/+^ spleen at different ages. All error bars indicate SD; N=4.

**
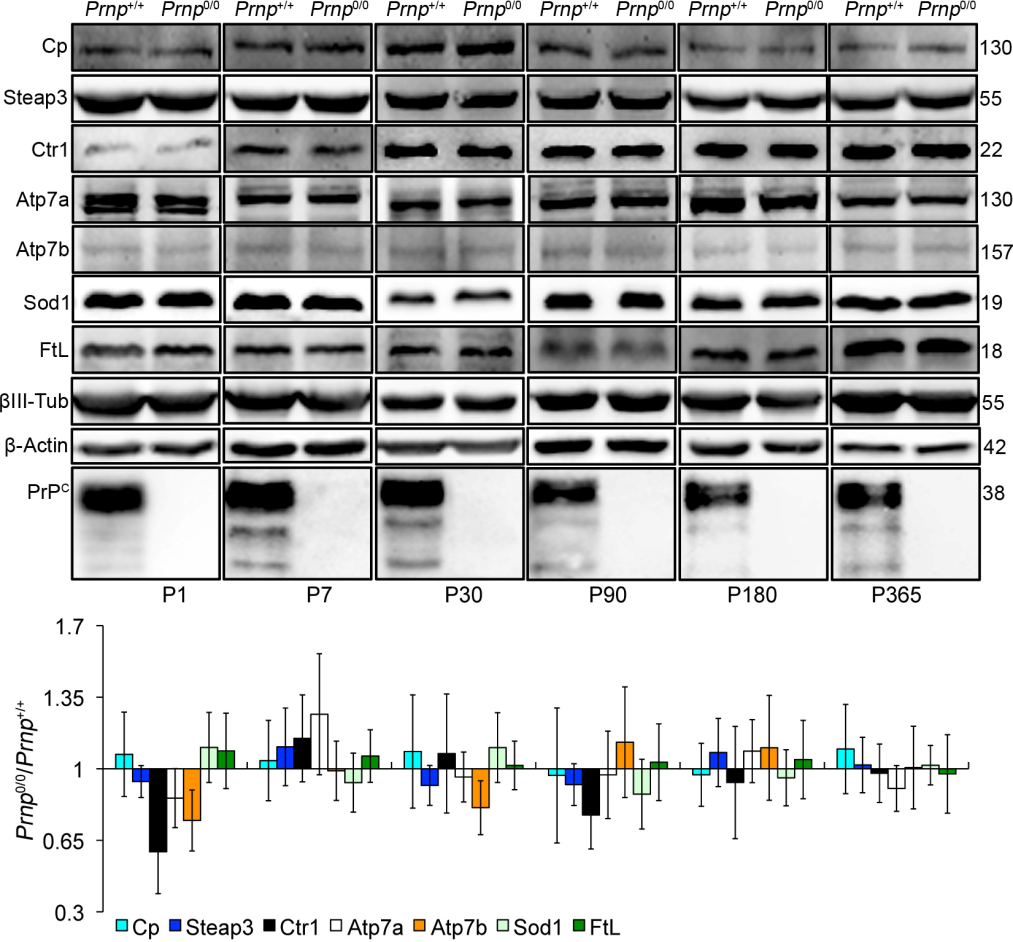
**

**Figure S6. Analysis of metal-binding protein expression in wild-type and PrP^C^-null mouse brain at different ages.**

In the upper part of the figure, representative Western blot images showing metal-binding protein levels in *Prnp*^0/0^ and *Prnp*^+/+^ brain samples. The constant level of the housekeeping proteins (β-III Tubulin and β-Actin) are also reported. In the lower part of the figure, the graph shows the up- or down-regulation of protein expression in *Prnp*^0/0^ samples compared to *Prnp*^+/+^, i.e. (*Prnp*^0/0^ protein OD/housekeeping OD)/ (*Prnp*^+/+^ protein OD/housekeeping OD). All error bars indicate SD; N=4 minimum.

**
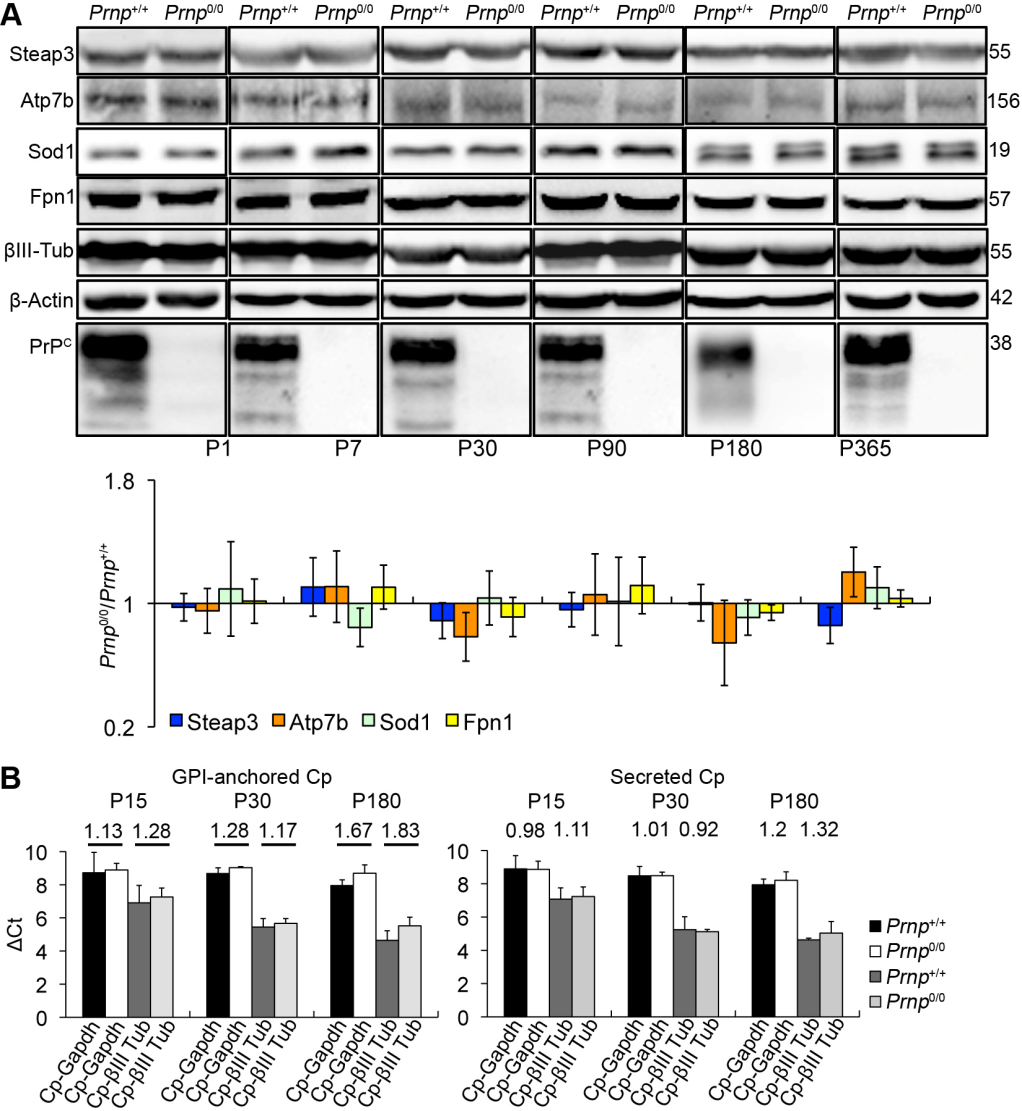
**

**Figure S7. Analysis of metal-binding protein expression in wild-type and PrP^C^-null mouse hippocampus at different ages.**

(**A**) In the upper part of the panel, representative Western blot images showing metal-binding protein levels in *Prnp*^0/0^ and *Prnp*^+/+^ brain samples. The constant level of the housekeeping proteins (β-III Tubulin and β-Actin) are also reported. In the lower part of the panel, the graph shows the up- or down-regulation of protein expression in *Prnp*^0/0^ samples compared to *Prnp*^+/+^, i.e. (*Prnp*^0/0^ protein OD/housekeeping OD)/ (*Prnp*^+/+^ protein OD/housekeeping OD). All error bars indicate SD; N=4 minimum. (**B**) Secreted and GPI-anchored Cp mRNA analysis by qRT-PCR in *Prnp*^0/0^ and *Prnp*^+/+^ liver at different ages (N=4). The graph shows the ΔCt values obtained by normalizing Cp Ct on two housekeeping genes (Gapdh and βIII-Tubulin). The numbers reported on the graph columns indicate the fold change values.

**References**

Al-Sa'doni, H.H., Megson, I.L., Bisland, S., Butler, A.R., and Flitney, F.W. (1997). Neocuproine, a selective Cu(I) chelator, and the relaxation of rat vascular smooth muscle by S-nitrosothiols. *Br J Pharmacol* 121**,** 1047-1050.

Chen, L., Dentchev, T., Wong, R., Hahn, P., Wen, R., Bennett, J., and Dunaief, J.L. (2003). Increased expression of ceruloplasmin in the retina following photic injury. *Mol Vis* 30**,** 151-158.

Livak, K.J., and Schmittgen, T.D. (2001). Analysis of relative gene expression data using real-time quantitative PCR and the 2(-Delta Delta C(T)) Method. *Methods* 25**,** 402-408.

Simonetti, M., Giniatullin, R., and Fabbretti, E. (2008). Mechanisms mediating the enhanced gene transcription of P2X3 receptor by calcitonin gene-related peptide in trigeminal sensory neurons. *J Biol Chem* 283**,** 18743-18752.

Stasi, K., Nagel, D., Yang, X., Ren, L., Mittag, T., and Danias, J. (2007). Ceruloplasmin upregulation in retina of murine and human glaucomatous eyes. *Invest Ophthalmol Vis Sci* 48**,** 727-732.
